# Automatic analysis of normative retinal oximetry images

**DOI:** 10.1371/journal.pone.0231677

**Published:** 2020-05-18

**Authors:** J. R. Harish Kumar, Chandra Sekhar Seelamantula, Ashwin Mohan, Rohit Shetty, T. J. M. Berendschot, Carroll A. B. Webers

**Affiliations:** 1 Department of Electrical Engineering, Indian Institute of Science, Bangalore, India; 2 Department of Electrical and Electronics Engineering, Manipal Institute of Technology, MAHE, Manipal, India; 3 Postgraduate Institute of Ophthalmology, Narayana Nethralaya, Bangalore, India; 4 University Eye Clinic Maastricht, Maastricht, The Netherlands; Swinburne University of Technology, AUSTRALIA

## Abstract

Retinal oximetry is an important screening tool for early detection of retinal pathologies due to changes in the vasculature and also serves as a useful indicator of human-body-wide vascular abnormalities. We present an automatic technique for the measurement of oxygen saturation in retinal arterioles and venules using dual-wavelength retinal oximetry images. The technique is based on segmenting an optic-disc-centered ring-shaped region of interest and subsequent analysis of the oxygen saturation levels. We show that the two dominant peaks in the histogram of the oxygen saturation levels correspond to arteriolar and venular oxygen saturations from which the arterio-venous saturation difference (AVSD) can be calculated. For evaluation, we use a normative database of Asian Indian eyes containing 44 dual-wavelength retinal oximetry images. Validations against expert manual annotations of arterioles and venules show that the proposed technique results in an average arteriolar oxygen saturation (*SatO*_2_) of 87.48%, venular *SatO*_2_ of 57.41%, and AVSD of 30.07% in comparison with the expert ground-truth average arteriolar *SatO*_2_ of 89.41%, venular *SatO*_2_ of 56.32%, and AVSD of 33.09%, respectively. The results exhibit high consistency across the dataset indicating that the automated technique is an accurate alternative to the manual procedure.

## 1. Introduction

The retina is a light-sensitive tissue layer at the posterior inside of the eye. Millions of nerve axons running all over the retina convert the incident light into neural signals, which are carried to the brain by the optic nerve for visual perception. Oxygen is important for normal functionality and metabolism in the retina. The retinal arteries carrying oxygenated hemoglobin (*HbO*_2_) enter the retina, and veins carrying deoxygenated hemoglobin (*Hb*) leave the retina through the optic nerve head. In a retinal fundus image, the optic nerve head appears as a disc, referred to as the optic disc (OD). The retina has the highest metabolic demand than any other tissue in the body [1]. Inadequate delivery and abnormal utilization of oxygen alter the normal functioning of the retina and trigger diseases such as diabetic retinopathy, glaucoma, age-related macular degeneration, retinal vein occlusions, and retinal detachment, leading to vision impairment [2–7]. Early detection of the pathological condition followed by suitable treatment may improve the retinal blood flow and oxygenation and restore vision [3–7].

**Fig 1:**
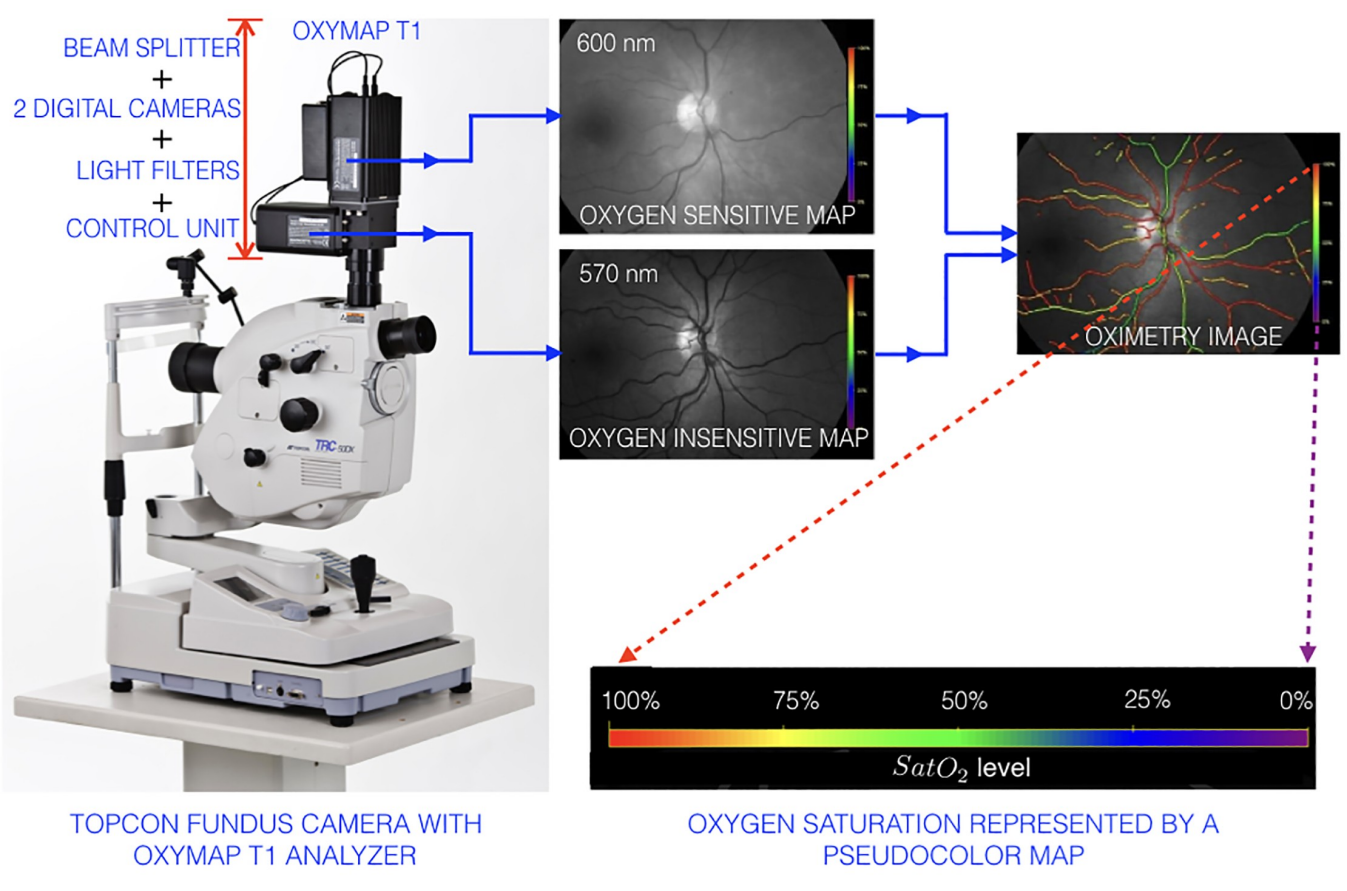
[Color online] the Oxymap T1 mounted on top of a Topcon TRC-50DX retinal fundus camera and an illustration of the principle of retinal oximetry [8].

The retinal fundus image provides a direct view of the vasculature and can be used to compute oxygen saturation levels. The quest for retinal metabolic analysis through measurement of oxygen saturation resulted in a non-invasive dual-wavelength spectrophotometric retinal oximeter [8, 9]. The Oxymap T1 (Oxymap, Reykjavik, Iceland) is a non-invasive dual-wavelength oximeter, which consists of an optical adapter, two high-resolution digital cameras, an image splitter, and two narrow band-pass filters. An Oxymap T1 mounted on top of a Topcon TRC-50DX (Topcon Corporation, Tokyo, Japan) fundus camera is shown in Fig 1.

In a dual-wavelength oximeter, retinal fundus images are simultaneously captured with two wavelengths of light, one at 600 nm, which is sensitive to oxygen saturation, and the other at 570 nm, which is not oxygen sensitive, but is used to calibrate the light intensity. The images are processed by the Oxymap Analyzer software to detect the blood vessels and estimate the light absorbance (optical density) at each point along the vessels for each wavelength and generate a pseudocolor map of the oxygen saturation [8]. The principle of retinal oximetry image acquisition and a representative image with the pseudocolor map is shown in Fig 1.

### 1.1 Prior Art

Hickam et al. [10, 11] were the first to propose non-invasive retinal oximetry using special filters. The objective of their study was to investigate retinal blood circulation and metabolism in humans. They developed techniques for the measurement of diameter changes in retinal vessels for various stimuli and also oxygen saturation of retinal venous blood using fundus images. Several authors have contributed to the analysis of retinal oxygen saturation under healthy and diseased conditions of the retina and also development of the multi-wavelength oximetry system. Dalori et al. [12] proposed a noninvasive spectrophotometric technique and determined the oxygen saturation after compensating for the effect of light scattering by the red blood cells. Sebag et al. [13] studied the effect of optic atrophy on retinal blood flow and oxygen saturation in humans. Denninghoff et al. [14] reported the oxygen saturation in retinal vessels during hypoxia. Tiedeman et al. [15] studied the retinal oxygen consumption in patients with diabetes. Beach et al. [16] proposed a method for noninvasive measurement of oxygen saturation using digital imaging techniques. They recorded images at 600 nm and 569 nm (oxygen-sensitive and oxygen-insensitive wavelengths, respectively) by using a modified fundus camera with an image splitter coupled to an 18-bit digital camera. Schweitzer et al. [17] considered wavelengths between 510 nm and 586 nm. They used the experimentally determined transmission spectra and the spectra of the internal reflection of saturated blood at a large number of wavelengths to calculate the oxygen saturation. Crittin et al. [18] developed a reflectance oximeter and showed that the optical density ratio could be used for relative oxygen saturation measurements. Harris et al. [19] reviewed the literature on the advancements in retinal oximetry methods until the year 2003. Hardarson et al. showed the reliability of retinal oximeter [20] and also analyzed the oxygen saturation under retinal vein occlusion [29] and diabetic retinopathy conditions [34]. Michelson et al. [21] and Olafsdottir et al. [22] examined the oxygen saturation in retinal arterioles and venules simultaneously by imaging spectrometry and measured the oxygen saturation in patients with glaucoma. Schweitzer et al. [23] showed that healthy subjects and diabetics in the early stages of diabetic retinopathy exhibit comparable changes of oxygen saturation during breathing of 100% oxygen. Kagemann et al. [24] proposed Fourier-domain optical coherence tomography to assess spectral oximetry. Three-dimensional disc-centered retinal tissue volumes were assessed in healthy subjects. In this study, a two-wavelength optical density ratio approach was employed. Johnson et al. [25] presented a snapshot imaging spectrometer that acquires a complete spatial-spectral image cube in approximately 3 ms from 450 nm to 700 nm with 50 bands. The setup coupled to a fundus camera gave a true color retinal image. Hammer et al. [26] used a fundus camera equipped with a special dual-wavelength transmission filter and a color charge-coupled device camera. Two monochromatic fundus images recorded at 548 nm and 610 nm were used for the analysis. Ramella-Roman et al. [27] introduced a multi-aperture camera system based on a lenslet array architecture and captured images in six spectral bands. They reported *in vivo* testing on healthy volunteers.

Hammer et al. [28] showed increased retinal venous oxygen saturation in diabetic retinopathy patients. Denninghoff et al. [30] reported the first noninvasive *in vivo* application of blue-green oximetry to retinal vessels using a modified confocal scanning laser ophthalmoscope. Li et al. [31] used an adaptive optics-based confocal scanning laser ophthalmoscope to measure oxygen saturation in small retinal vessels. Images with a diameter smaller than 50 microns were recorded at 680 nm (oxygen-sensitive) and 796 nm (oxygen-insensitive) wavelengths. They showed that the oxygen saturation in the artery is higher than that in the vein and also that the oxygen saturation in small vessels can be affected by the metabolic activity in the retina. Mordant et al. [32, 33] used images obtained at wavelengths 500 nm and 650 nm (oxygen-sensitive and oxygen-insensitive, respectively) acquired from a hyper-spectral fundus camera and analyzed them with an oximetry model to measure oxygen saturation.

There have been a few manual attempts to measure retinal oximetry values from fundus images [17, 20]. According to the study by Schweitzer et al. [17], the mean oxygen saturation levels for retinal arterioles and venules in healthy individuals are 92.2% and 57.9%, respectively. Oximetry analysis revealed that the oxygenation levels change in many retinal pathologies such as glaucoma [21, 22], diabetic retinopathy [23, 28, 34], retinal vein occlusions [5, 29], and systemic hypoxemia [35]. Jani et al. [36], Geirsdottir et al. [9], and Mohan et al. [2] have established normative databases using the Oxymap T1 retinal oximeter. Their study on the variability of oxygen saturation in healthy individuals revealed that the age, vessel diameter, and ocular perfusion pressure are significant factors that influence the saturation. The findings were based on manual marking of the arterioles and venules in the OD-centered ring-shaped region of interest on the dual-wavelength retinal oximetry images [2, 9].

The current practice to calculate oxygen saturation from the fundus image requires considerable manual effort. To start with, one must manually outline the OD and segment a ring-shaped region of interest that is concentric with the OD. Subsequently, the arterioles and venules have to be identified, and the oxygen saturation level and arterio-venous saturation difference (AVSD) has to be estimated. This procedure is not only tedious but also introduces subjectivity. Hence, there is a need for an automated and consistent analysis methodology. To the best of our knowledge, a computer-aided technique for solving the problem has not been reported in the literature although there is a pressing clinical need. The objective of this paper is to fill this gap.

## 2. Methods

We have created a normative retinal oximetry image database of Asian Indian population using the Oxymap-Topcon duo [2]. We propose a fully automated technique for performing retinal arteriolar and venular oxygen saturation measurements as an efficient and robust alternative to manual assessment [2,9]. The reliability and accuracy of the proposed technique are also measured considering manual assessment as the baseline.

### 2.1 Automated segmentation of the region of interest

We segment the region of interest based on the *active-disc method* [37] introduced recently for the segmentation of optic disc and cup and subsequent measurement of glaucoma specific parameters such as the cup-to-disc ratio and the rim-to-disc ratio [38]. The active-disc method is motivated by the techniques developed by Pediredla et al. [39] and Thévenaz et al. [40]. The method is iterative and performs shape-constrained segmentation. In our analysis, the shape is constrained to be a circle. The initialization is automatic and is based on normalized cross-correlation. The parameters are the coordinates of the center of the disc and the radius, which are optimized to minimize a locally computed energy function. The optimization is carried out efficiently using gradient-descent technique [41] and Green’s theorem [42].

**Fig 2:**
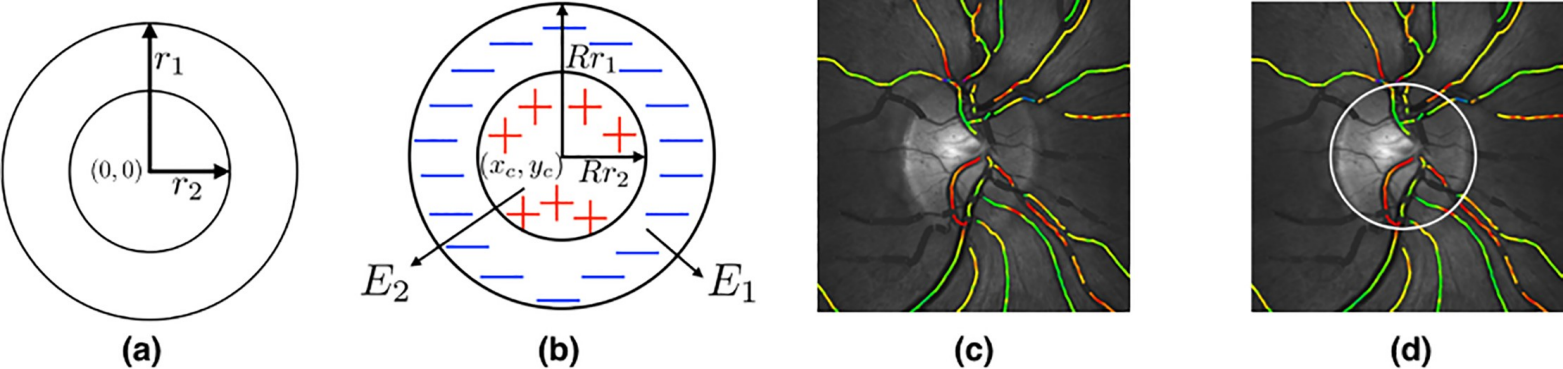
[Color online] (a) A circular template; (b) Circular active disc; (c) Optic disc image in dual-wavelength oximetry; and (d) Optimal active disc fit to the optic disc.

The *active-disc* comprises two concentric circles centered at the origin and parameterized as follows:

(xi(t)yi(t))=(ricostrisint), (1)

for *i* = 1,2, and ∀*t*∈(0,2*π*], where *r*_1_ and *r*_2_ denote the radii of the outer and inner circles, respectively, which are set to 1 and 1/√2, respectively. An example of such a template is shown in Fig 2(A). The concentric circles with isotropic scaling and translation are given by

(XiYi)=R(xiyi)+(xcyc), (2)

where *i* = 1,2, and (*X*_1_,*Y*_1_), and (*X*_2_,*Y*_2_) denote the outer and inner boundaries, respectively; *R* represents the scale parameter, and (*x*_*c*_,*y*_*c*_) are the translational parameters, amounting to a total of three degrees of freedom. For brevity of notation, we drop the variable *t* and replace (*x*_*i*_(*t*),*y*_*i*_(*t*)) and (*X*_*i*_(*t*),*Y*_*i*_(*t*)) with (*x*_*i*_,*y*_*i*_) and (*X*_*i*_,*Y*_*i*_), respectively. The active disc is illustrated pictorially in Fig 2(B). The active-disc energy is a normalized contrast function, which considers the area inside the inner disc as the foreground and the annular region as the background. For an image *f*, let *R*_1_ and *R*_2_ denote the regions enclosed by the outer and inner discs, respectively. The active-disc energy is chosen to be

$$\begin{array}{l} E=\frac{1}{R^{2}}\left(\iint_{R_{1}\backslash R_{2}}\;f(X,Y)dXdY-\iint_{R_{2}}\;f(X,Y)dXdY\right),\\ \;=\frac{1}{R^{2}}\left(\underset{E_{1}}{\underset{︸}{\iint_{R_{1}}\;f(X,Y)dXdY}-2}\underset{E_{2}}{\underset{︸}{\iint_{R_{2}}\;f(X,Y)dXdY}}\right),\\ \;=\frac{1}{R^{2}}(E_{1}-2E_{2}), \end{array}$$ (3)

where *E*_1_ and *E*_2_ are the image energies in the regions *R*_1_ and *R*_2_, respectively. Minimizing the active-disc energy gives a tight fit contour of the optic disc. Fig 2(C) and 2(D) show an example of the OD in a dual-wavelength oximetry image and the optimal active disc fit to the OD boundary, respectively.

We perform optimization using gradient-descent technique [41], which is a first-order approach. One starts with an initial guess *P*_0_, where *P* is a generic variable that is used to denote the parameters *R*,*x*_*c*_, and *y*_*c*_, and updates *P* as follows:

$$P_{n+1}=P_{n}-\gamma_{n}\nabla E[P_{n}],$$

where *E*[*P*_0_]≥*E*[*P*_1_]≥*E*[*P*_2_]…, and ∇ denotes the gradient. The parameter *γ*_*n*_>0 is the step-size parameter.

The gradient-descent technique requires partial derivatives of the energy function. Since the integrals are two-dimensional and the contours are closed, one could compute the partial derivatives efficiently using Green’s theorem [42]. In our optimization, we need partial derivatives of the energy function with respect to the parameters *R*,*x*_*c*_, and *y*_*c*_. We show the calculations for one of the parameters. Applying Green’s theorem to *E*_2_ gives

$$E_{2}=\oint_{R_{2}}f^{X}dY=-\oint_{R_{2}}f^{Y}dX,$$ (4)

where $f^{X}(X,Y)=\int_{-\infty }^{X}f(\zeta ,Y)d\zeta$ and $f^{Y}(X,Y)=\int_{-\infty }^{Y}f(X,\zeta )d\zeta$. *E*_2_ is a function of (*X*,*Y*), which are functions of the parameters of the disc. The partial derivative of *E*_2_ with respect to *R* is given by

$$\frac{\partial E_{2}}{\partial R}=\frac{\partial E_{2}}{\partial X}\frac{\partial X}{\partial R}+\frac{\partial E_{2}}{\partial Y}\frac{\partial Y}{\partial R}.$$ (5)

Substituting (4) in (5) and simplifying gives

$$\begin{array}{l} \frac{\partial E_{2}}{\partial R}=\oint_{R_{2}}\frac{\partial f^{X}}{\partial X}\frac{\partial X}{\partial R}dY-\oint_{R_{2}}\frac{\partial f^{Y}}{\partial Y}\frac{\partial Y}{\partial R}dX,\\ \;=\frac{R}{2}\int_{t=0}^{2\pi }f(X_{2},Y_{2})dt. \end{array}$$ (6)

The partial derivative of the energy *E*_1_ with respect to *R* can be found similarly:

$$\begin{array}{l} \frac{\partial E_{1}}{\partial R}=\oint_{R_{1}}\frac{\partial f^{X}}{\partial X}\frac{\partial X}{\partial R}dY-\oint_{R_{1}}\frac{\partial f^{Y}}{\partial Y}\frac{\partial Y}{\partial R}dX,\\ \;=R\int_{t=0}^{2\pi }f(X_{1},Y_{1})dt. \end{array}$$ (7)

The partial derivative of the energy *E* with respect to *R* can be obtained as follows:

$$\frac{\partial E}{\partial R}=\frac{1}{R^{2}}\left(\frac{\partial }{\partial R}(E_{1}-2E_{2})\right)-\frac{2}{R^{3}}(E_{1}-2E_{2}).$$ (8)

Substituting (6) and (7) in (8) and simplifying, we get

$$\frac{\partial E}{\partial R}=\frac{1}{R}\left(\int_{t=0}^{2\pi }f(X_{1},Y_{1})dt-\int_{t=0}^{2\pi }f(X_{2},Y_{2})dt-2E\right).$$ (9)

The partial derivatives of the energy *E* with respect to the coordinates of the center of the disc can be found as follows:

$$\frac{\partial E}{\partial x_{c}}=\frac{1}{R^{2}}\left(\int_{t=0}^{2\pi }(\sqrt{2}\;f\;(X_{1},Y_{1})\;dt-2f\;(X_{2},Y_{2}))\;cost\;dt\right),$$ (10)

$$\frac{\partial E}{\partial y_{c}}=\frac{1}{R^{2}}\left(\int_{t=0}^{2\pi }(\sqrt{2}\;f\;(X_{1},Y_{1})\;dt-2f\;(X_{2},Y_{2}))\;sint\;dt\right).$$ (11)

**Fig 3:**
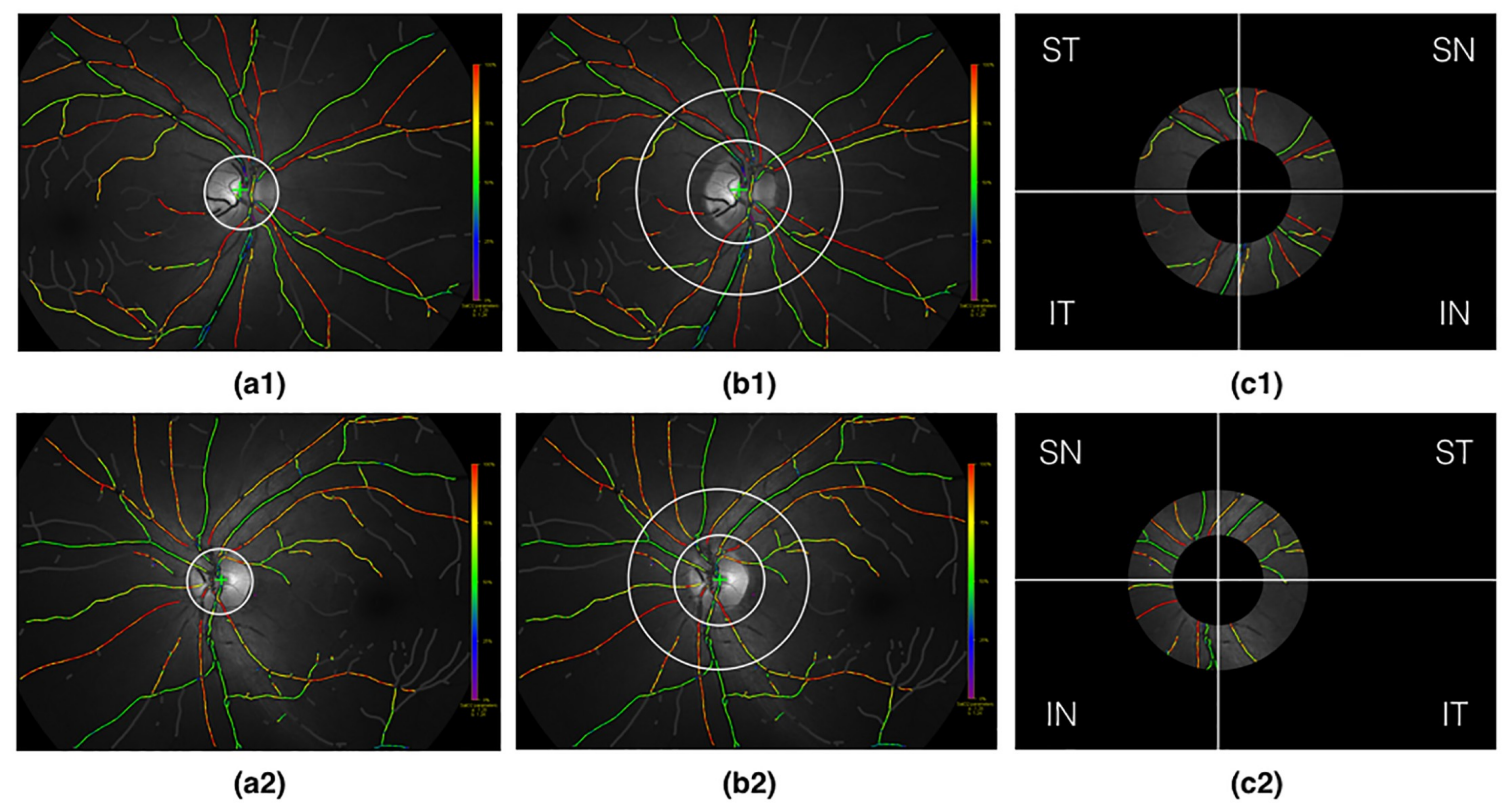
Illustration of OD-centered ring-shaped region-of-interest segmentation for oximetry analysis on the right eye (first row) and left eye (second row). (a1)-(a2): Detection (green +) and segmentation of the optic disc (white circle); (b1)-(b2): Delineation of the ring-shaped region of interest (white concentric circles); (c1)-(c2): Segmentation of the ring-shaped region of interest and partitioning into supero-temporal (ST), supero-nasal (SN), infero-nasal (IN), and infero-temporal (IT) quadrants.

After accurate localization and segmentation of the OD, we follow the protocol adopted by Mohan et al. [2] and Geirsdottir et al. [9] for segmenting the OD-centered ring-shaped region of interest and subsequent oxygen saturation analysis. The region consisting of the annulus between two circles of radius (50 + *R*) pixels and 2(50 +*R*) pixels (1 pixel = 9 microns) concentric with the OD is selected for computing the oxygen saturation. The region is partitioned into supero-temporal (ST), supero-nasal (SN), infero-nasal (IN), and infero-temporal (IT) quadrants. Fig 3 illustrates the complete procedure.

### 2.2 Algorithm for measurement of oxygen saturation

The oxygenated blood enters the eye through the central retinal arteriole and gets divided into four main branches, which are the major vessels and have thick walls. The blood then flows through the smaller arterioles and the capillary bed where oxygen exchange takes place. The blood then enters the smaller venules and finally the central retinal venule through the four larger venules. These larger venules much like the arterioles, have done oxygen exchange due to the thick walls and as a result, the oxygen saturation would be relatively stable in the larger vessels where fundus oximetry measurements are made.

Oxygen saturation (*SatO*_2_) is the percentage of hemoglobin that is bound to oxygen [12] and is measured as follows:

$$SatO_{2}=\frac{HbO_{2}}{HbO_{2}+Hb}\times 100.$$

**Fig 4:**
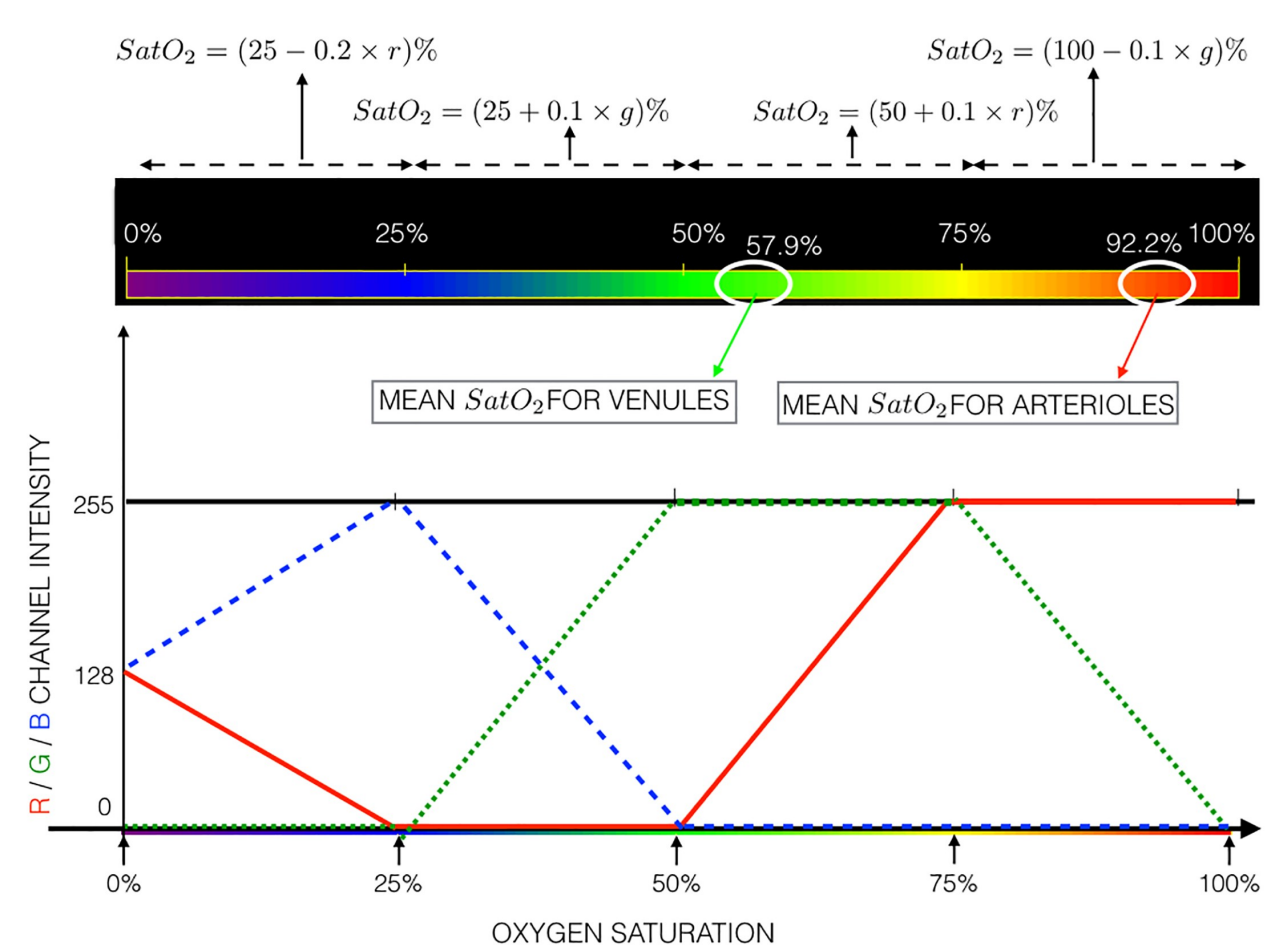
[Color online] Interpretation of the pseudocolor map for *SatO*_2_ in retinal arterioles and venules together with their mean values in healthy individuals according to the clinical study reported in [17]. The relationship between oxygen saturation and the corresponding red/green/blue component contribution for pixel color is also shown.

Since about 98.5% of the oxygen carried in blood is bound to hemoglobin, *SatO*_2_ is an accurate measure of the amount of oxygen in blood [43–45]. The principle of retinal oximetry is based on differential light absorption of *HbO*_2_ and *Hb* [45]. Fully oxygenated blood appears as bright red and deoxygenated blood reflects in the green to violet color bands. Various oxygen saturation levels correspond to different colors from red to the violet band of the spectrum [45]. The mean oxygen saturation for retinal arterioles and venules in healthy individuals is 92.2% and 57.9%, respectively [17] (cf. Fig 4). The pseudocolor maps could be used to assess the saturation values. The resulting saturation values have a bimodal distribution, one corresponding to the arterioles and the other corresponding to the venules.

The relationship between *SatO*_2_ and the respective red/green/blue component for pixel intensity is shown in Fig 4 and can be specified by the following:

Condition-1:

$$\mathrm{if}\;r=255,\;g\neq 255,\;b=0;\;\mathrm{then}\;\mathrm{Sat}O_{2}=100-0.1\times g;\;\mathrm{and}$$

$$\mathrm{if}\;r\neq 255,\;g=255,b=0;\;\mathrm{then}\;\mathrm{Sat}O_{2}=50+0.1\times r.$$

Condition-2:

$$\mathrm{if}\;r=0,\;g\neq 0,\;b\neq 0;\;\mathrm{then}\;\mathrm{Sat}O_{2}=25+0.1\times g.$$

Condition-3:

$$\mathrm{if}\;r\neq 0,\;g=0,\;b\neq 0;\;\mathrm{then}\;\mathrm{Sat}0_{2}=25-0.2\times r.$$

**Fig 5:**
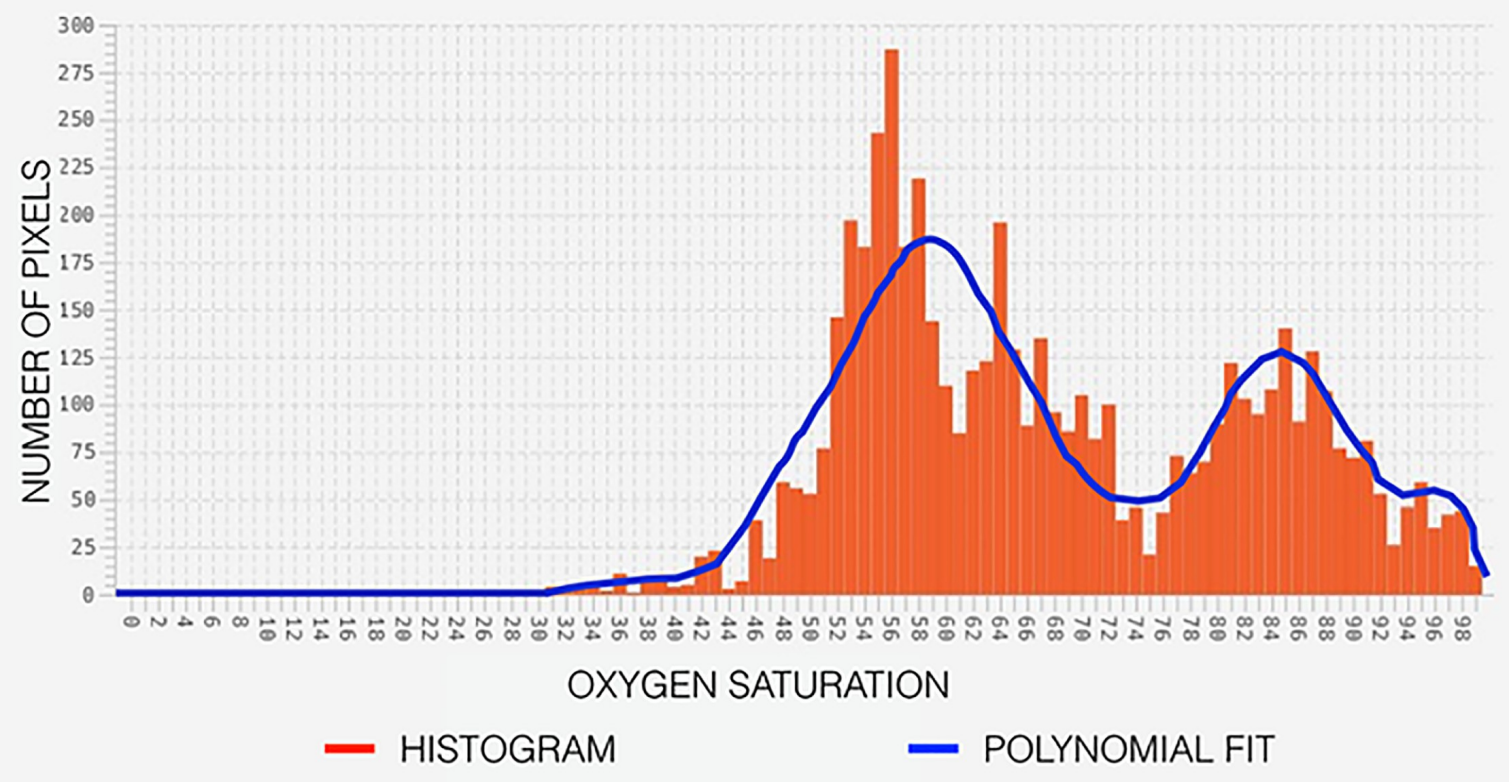
[Color online] A histogram representing oxygen saturation in the region of interest and a bimodal curve fit to it.

A histogram of oxygen saturation levels for an example oximetry image is shown in Fig 5. The histogram has a bimodal distribution with peaks very close to the *SatO*_2_ in the venules and arterioles. The bimodal peaks are identified using a least-squares polynomial fit of order twelve. The two dominant peaks represent the *SatO*_2_ in the venules and arterioles, respectively. We follow the same procedure for the ST, SN, IN, and IT quadrant-based oxygen saturation analysis.

## 3. Results and discussion

We have created a normative database [2] of retinal oximetry in Asian Indian eyes for validating the proposed technique. The ground truth has been obtained by manually marking all the vessels in the region of interest that are above the cut-off of 8 pixels (equivalently, 72 microns) in diameter. The manual method accounts for the values in all the pixels in a selected vessel and averages them out. This is prone to error and skewing of the mean due to extremes. The algorithm reverse-calculates the values given on the exported retinal oximetry image with the help of the pseudocolor map. The bimodal fit on the histogram of oxygen saturation levels gives an accurate saturation estimate.

**Fig 6:**
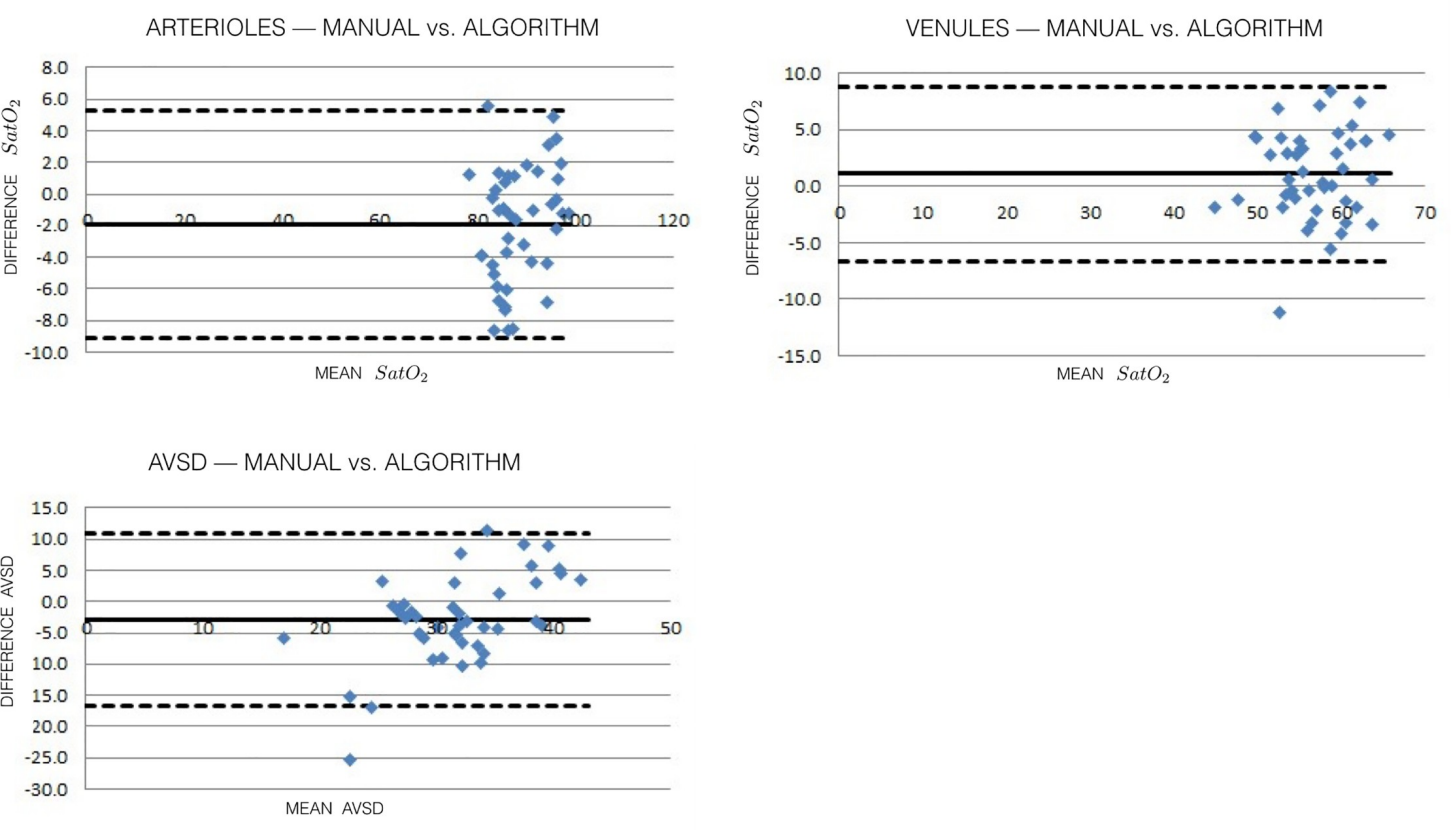
(a): [Color online] Bland-Altman plot for comparing the algorithm-derived arteriolar oxygen saturation levels versus manual measurements for 44 images. (b): [Color online] Bland-Altman plots for comparing the algorithm-derived venular oxygen saturation levels versus manual measurements for 44 images. (c): [Color online] Bland-Altman plots for comparing the algorithm-derived AVSD versus manual measurements for 44 images.

**Fig 7:**
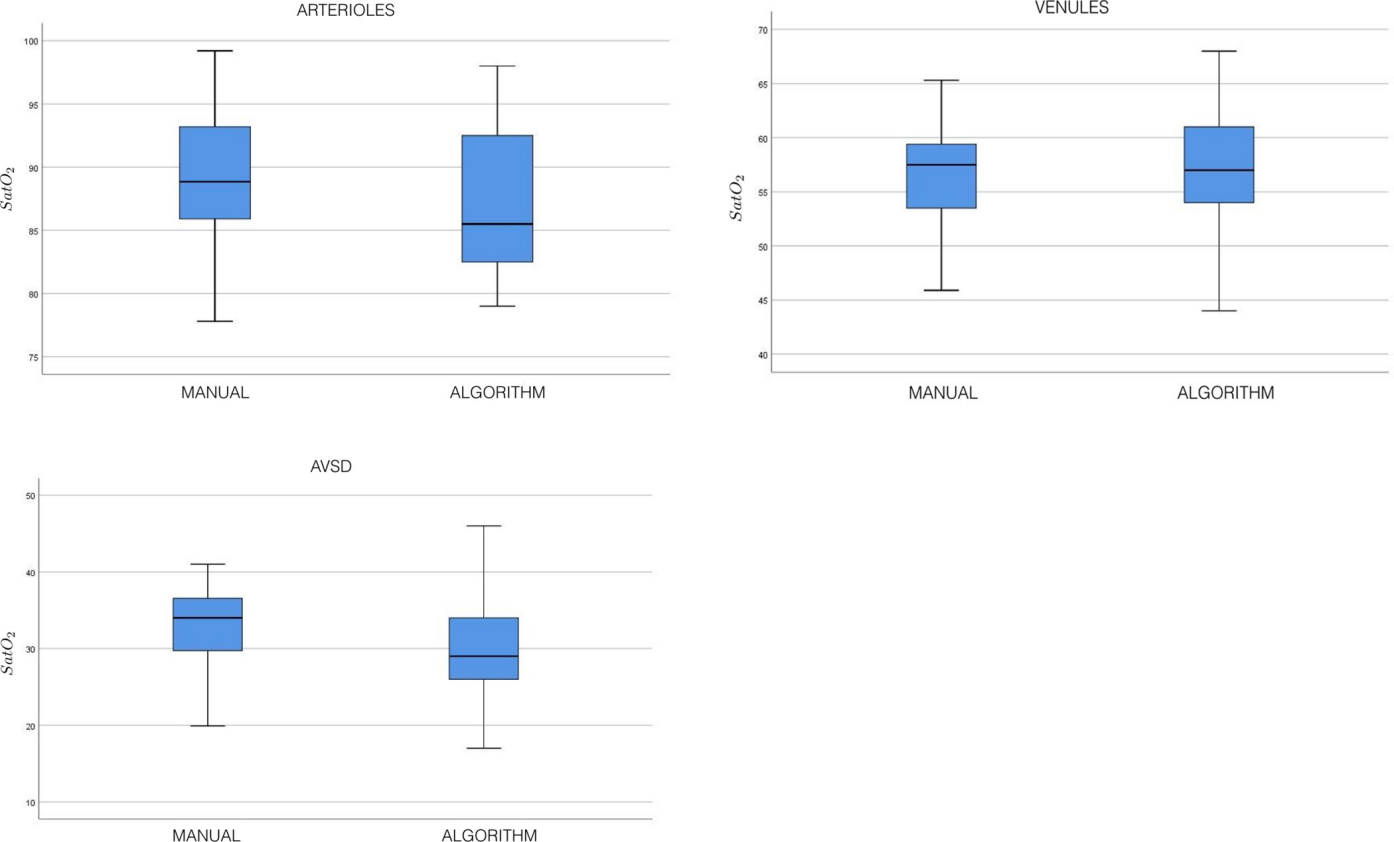
(a): [Color online] Boxplots for comparing the algorithm-derived arteriolar oxygen saturation levels versus manual measurements for 44 images. (b): [Color online] Boxplots for comparing the algorithm-derived venular oxygen saturation levels versus manual measurements for 44 images. (c): [Color online] Boxplots for comparing the algorithm-derived AVSD versus manual measurements for 44 images.

A total of 44 retinal oximetry images of healthy individuals of Asian Indian origin were used in the study. The bimodal peaks are identified and arteriolar and venular saturations and AVSD values are automatically calculated. In Table 1, we compare the average of the manually obtained values against those computed by the proposed algorithm. We observe that the algorithm results are comparable to the manual measurement. We use the intraclass correlation coefficient (ICC) as a measure of reliability of the algorithm estimate of oxygen saturation in comparison with that of the ground-truth. We present the ICC for single and average measures with 95% confidence interval (CI). For arteriolar oxygen saturation, the ICC showed a good agreement for single measures (0.66; 95% CI: 0.51 to 0.78) and excellent agreement for average measures (0.85; 95% CI: 0.76 to 0.92). For venular oxygen saturation, the ICC showed a good agreement for single measures (0.61; 95% CI: 0.45 to 0.74) and excellent agreement for average measures (0.82; 95% CI: 0.71 to 0.90). For the AVSD, the ICC showed fair agreement for single measures (0.47; 95% CI: 0.29 to 0.64) and good agreement for average measures (0.72; 95% CI: 0.55 to 0.84). We compare the manual and algorithm measurements of arteriolar and venular oxygen saturation using the Bland-Altman difference plots. In this method, one plots the differences between the manual and algorithm results against the averages of the two techniques. The Bland-Altman plots for measured oxygen saturation in arterioles, venules, and AVSD versus manual assessment are shown in Fig 6(A), 6(B) and 6(C), respectively. The plots show excellent agreement between the manual and algorithm results. There is only one estimate outside the limits of agreement for arteriolar and venular oxygen saturation and AVSD. The boxplots for the measured oxygen saturation in arterioles, venules, and AVSD versus manual assessment are shown in Fig 7(A), 7(B) and 7(C), respectively. The boxplots do not indicate the presence of outliers in the estimated oxygen saturation. The automated oximetry analysis on the complete ring-shaped ROI and all the four quadrants (ST, SN, IN, and IT) is provided in Table 1. A comparison of algorithm results for arteriolar and venular oxygen saturation and AVSD with that of the ground-truth for each quadrant is also provided in Table 1. The results show that the automated analysis exhibits a high degree of agreement with manual measurements. The standard deviations between the ground-truth and algorithm determined arteriolar, venular oxygen saturation, and AVSD for the ring-shaped ROI over 44 images are provided in Table 2.

**Table 1:** Comparison of estimated oxygen saturation versus the ground-truth. The numbers reported are obtained after averaging over 44 images. ST: supero-temporal; SN: supero-nasal; IN: infero-nasal; IT: infero-temporal.

	Region of interest			Quadrant-Arteriolar				Quadrant-Venular			
	Arterioles	Venules	AVSD	ST	SN	IN	IT	ST	SN	IN	IT
**Ground-truth**	89.41	56.32	33.09	88.00	93.50	91.40	85.50	55.90	58.70	60.10	51.10
**Algorithm**	87.48	57.41	30.07	86.06	92.90	92.10	87.55	55.30	63.06	61.20	52.30

**Table 2:** Comparison of standard deviation (computed from 44 images) of estimated oxygen saturation and the ground-truth for a ring-shaped ROI.

	Region of interest		
	Arterioles	Venules	AVSD
**Ground-truth**	5.15	4.73	4.51
**Algorithm**	6.13	5.13	6.09

From a clinical perspective, this study is important, because it shows that the algorithm could be used in place of manual measurements. It has multiple advantages. First, it is automatic and fast thus saving time and effort. Second, a trained grader would not be needed to measure the oxygen saturation from an oximetry image. Third, it would eliminate inter-observer variability as different observers may choose different vessel segments giving rise to different saturations. With the help of an automated technique, oximetry measurements can be standardized. Finally, since the proposed approach considers the bimodal peaks, it would not be affected by extreme values.

The supporting material available online [46] comprises the ImageJ plugin [47, 48] for implementing the technique presented in this paper, exemplar oximetry images, and a video demonstrating the functionality of the plugin.

## 4. Conclusions

We have developed an automated, reliable, and accurate technique for performing retinal arteriolar and venular oxygen saturation measurements as an efficient alternative to manual or semi-automated procedures. The segmentation of OD and subsequent extraction of ring-shaped region of interest is performed using the active-disc technique. The oxygen saturation level estimated is on par with that obtained by manual assessment. A bimodal fit on the histogram of the oxygen saturation levels showed prominent peaks corresponding to the venular and arteriolar oxygen saturations. The technique was validated on a normative database of Asian Indian eyes containing 44 retinal oximetry images. The validation resulted in an average arteriolar and venular oxygen saturation of 87.48% and 57.41%, respectively, and AVSD of 30.07%. The results are close to those obtained from manual procedures and are also consistent across the dataset.

## Supporting information

S1 Data (ZIP)
