# Supplementary figures and images for: Automatic analysis of normative retinal oximetry images
Source: PLoS One. 2020 May 18;15(5):e0231677. doi: 10.1371/journal.pone.0231677 (PMC7233590; doi:10.1371/journal.pone.0231677)

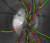

Supplement: S1 Data — (ZIP) [file pone.0231677.s001.zip › Supporting Material/Template_Image/OxiTemplate_OD.tif]

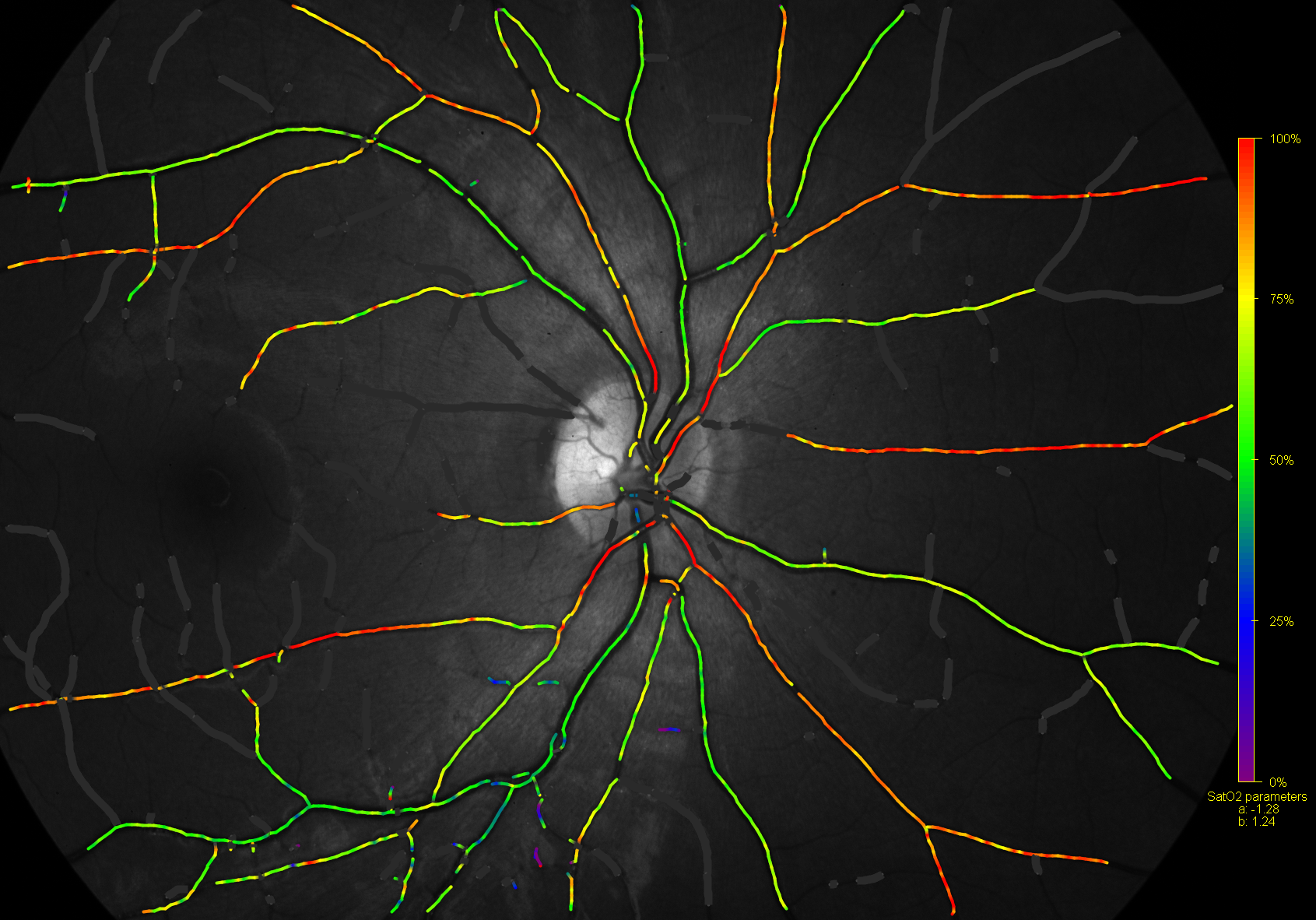

Supplement: S1 Data — (ZIP) [file pone.0231677.s001.zip › Supporting Material/Test_Oximetry_Images/NB01 OD.tif]

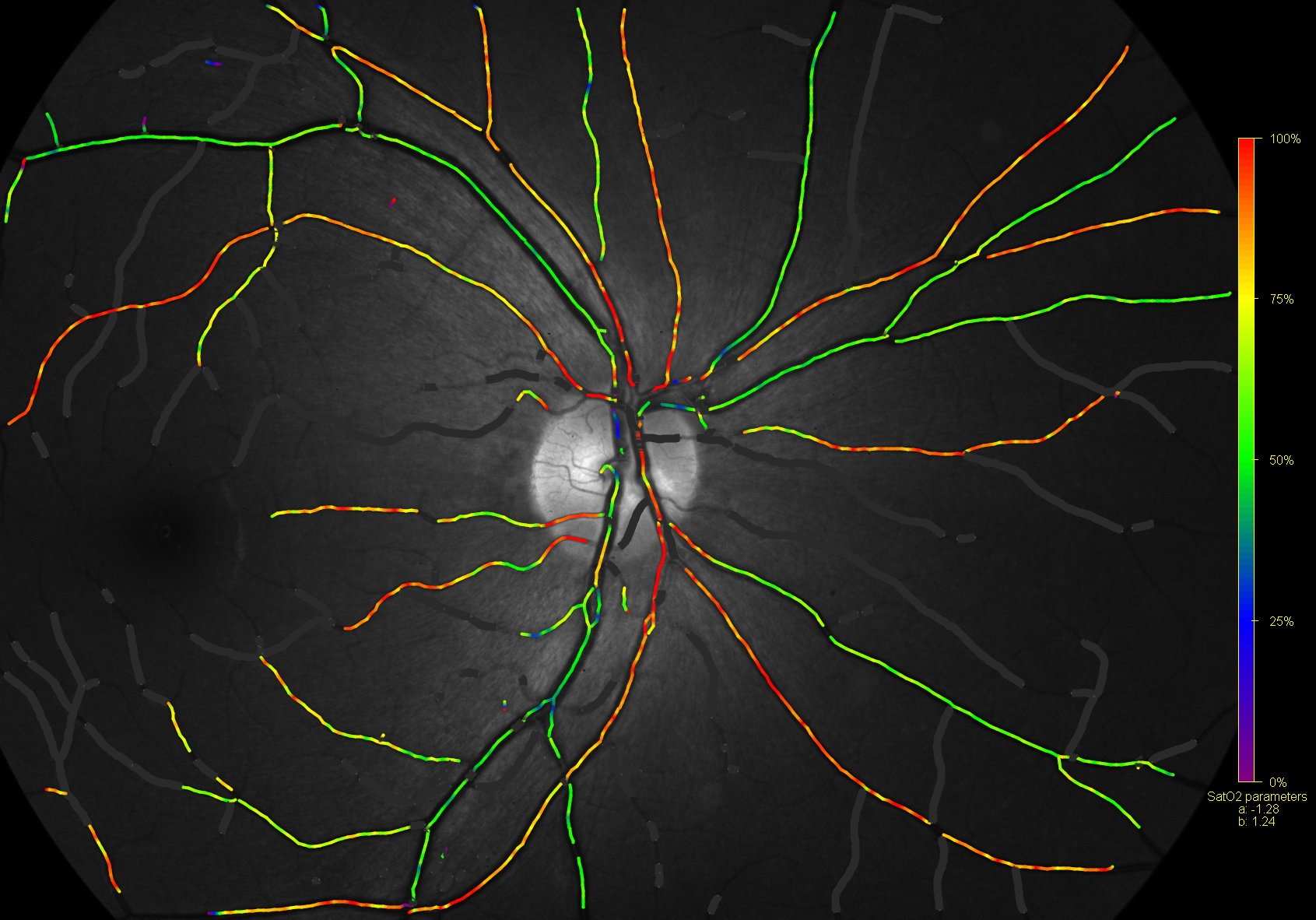

Supplement: S1 Data — (ZIP) [file pone.0231677.s001.zip › Supporting Material/Test_Oximetry_Images/NB03 OD.tif]
